# The Mitigating Effects of Perilla Leaf Essential Oil on the Phytotoxicity of Fenoxaprop-P-Ethyl in Rice Seedlings

**DOI:** 10.3390/plants13202946

**Published:** 2024-10-21

**Authors:** Jiuying Li, Yinghui Zhu, Lanlan Sun, Hongle Xu, Wangcang Su, Fei Xue, Chuantao Lu, Wenwei Tang, Renhai Wu

**Affiliations:** 1 Henan Key Laboratory of Crop Pest Control, Institute of Plant Protection, Henan Academy of Agricultural Sciences, Zhengzhou 450002, China; lijiuying90@163.com (J.L.); sunjgs@126.com (L.S.); xuhongle86@126.com (H.X.); suwangcang@126.com (W.S.); luchuantao@yeah.net (C.L.); 2 National Demonstration Center for Experimental Plant Science Education, Guangxi Key Laboratory of Agro-Environment and Agric-Product Safety, College of Agriculture, Guangxi University, Nanning 530004, China; wenweitg@163.com

**Keywords:** rice, fenoxaprop-P-ethyl, perilla leaf essential oil, natural safener, 2-hexanofuran

## Abstract

Fenoxaprop-P-ethyl (FE) can effectively control weeds in rice fields, but it has been found to cause phytotoxicity in rice. In this study, the phytotoxicity of FE was mitigated by perilla leaf essential oil (PEO) in rice seedlings. The injury recovery rates (IRRs) for shoot length and fresh weight treated with 800 mg/L of PEO were 101.51% and 99.05%, respectively. Moreover, the damage of *s*-metolachlor and pretilachlor was also alleviated when co-applied with 800 mg/L PEO; the IRR of *s*-metolachlor phytotoxicity was 26.07% and 27.34%, respectively, and the IRR of pretilachlor phytotoxicity was 127.27% and 124.39%, respectively. However, PEO had no significant effect on the phytotoxicity of pinoxaden, mesotrione, penoxsulam, mesosulfuron-methyl, and nicosulfuron. The results of GC–MS analysis showed that a total of 23 components were detected in PEO, among which linalool (36.49%), linalyl formate (26.96%), α-terpineol (10.63%), 2-hexanoylfuran (5.81%), geranyl acetate (4.13%), and neryl acetate (2.30%) were the primary components. Among them, 2-hexanoylfuran was the most effective component to alleviate FE damage, for which the IRR of shoot length and fresh weight was 73.17% and 73.02%, respectively, followed by the geranyl acetate, for which the IRR was 72.32% and 60.56%, respectively, and neryl acetate, for which the IRR was 65.28% and 58.11%, respectively. Furthermore, the application of 50 mg/L of 2-hexanofuran significantly improved the tolerance of shoot length and fresh weight to FE stress by factors of 5.32 and 5.35, respectively. This research demonstrates that PEO and 2-hexanoylfuran have the potential to serve as natural safeners to reduce phytotoxicity.

## 1. Introduction

Safeners are crucial agricultural compounds that enhance the selectivity of herbicides between crops and weeds to protect crops from the damaging effects of herbicides, which have been widely used to protect crops such as rice, corn, wheat, soybeans, cotton, and other crops in agricultural production [1]. However, the prolonged use of commercial safeners has exposed some of them to higher environmental pollution risks within agricultural water systems, thereby diminishing their safety for aquatic organisms and mammals [2,3,4]. Moreover, natural safeners hold greater research and application value in agricultural production due to their lower risk of environmental pollution and enhanced safety for mammals compared to synthetic safeners [5,6]. Plant essential oils comprise a diverse array of biochemically active secondary metabolites synthesized by plants [7]. These naturally occurring bioactive compounds, characterized by their relatively simple structures and ease of modification, represent valuable resources for the advancement of novel natural safeners [6,8,9]. At present, more than ten natural products with safener activities have been screened, such as z-ligustride, sanshools, bergapten, and isopimpinellin, which can alleviate the damage of herbicides in rice plants [5,6].

Perilla (*Perilla frutescens* L.) leaves, as an herbal medicine, showed strong anti-inflammatory, anticancer, and antioxidant activities, and its extract, perilla leaf essential oil (PEO), has many similar biological functions [10,11,12,13]. PEO also has excellent antibacterial activity and insecticidal activity, and can be used as a green pesticide in the field of agriculture, such as to protect against *Botrytis cinerea* during the storage of strawberries and to protect against *Cacopsylla chinensis* in pear orchards [14,15]. However, it has not been reported as an herbicide safener in agricultural production.

Rice (*Oryza sativa* L.) is a crucial agricultural commodity globally, functioning as the primary source of sustenance for more than fifty percent of the world’s population [16,17]. However, high weed infestation is a significant factor contributing to the loss of rice yield [18,19]. The main method for weed control is using chemical herbicides [20]. Fenoxaprop-P-ethyl (FE) belongs to the aryloxyphenoxypropionate (APP) herbicide class, which works by inhibiting acetyl-CoA carboxylase (ACCase) to prevent fatty acid biosynthesis in weeds [21]. It can effectively control common malignant weeds, such as *Echinochloa crusgalli* L., *Eleusine indica* L., *Digitaria sanguinalis* L., and *Leptochloa chinensis* L., and has broad application prospects [22]. Nonetheless, it has been observed that FE can readily induce phytotoxicity in rice seedlings, resulting in a substantial reduction in rice yield [23,24]. This phenomenon poses a considerable constraint on the utilization of FE in agricultural practices.

In this study, we evaluated the mitigating effect of PEO on the damage caused by herbicides in rice and analysed the primary active components of PEO using gas chromatography–mass spectrometry (GC–MS). The main components of PEO were assessed for biological activity to identify the compounds with the strongest mitigation potential. This study presents an innovative approach for the rational application of FE in rice cultivation, and presents a new lead compound for the development of innovative safeners.

## 2. Results

### 2.1. Chemical Composition of PEO

**Figure 1:**
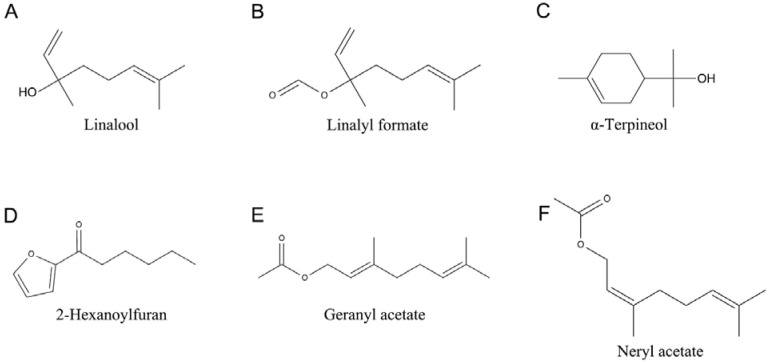
The structural formulas of the main substances of PEO. (**A**) Linalool; (**B**) Linalyl formate; (**C**) α-Terpineol; (**D**) 2-Hexanoylfuran; (**E**) Geranyl acetate; (**F**) Neryl acetate.

The GC–MS method was utilized to analyse the PEO, and the peak area percentage was employed as an indicator of the relative concentration. A total of 23 compounds were identified, constituting 97.95% of the PEO. The total ions chromatograph map of PEO is presented in Figure S1, and the MS spectra of these components, as well as the MS spectra from the NIST11s.lib database, are presented in Figure S2(1-23), in the Supplementary Materials. Table 1 presents the retention time and relative content of each compound. The results indicated that a total of six components had a relative content exceeding 2% in PEO, and these compounds are linalool (36.49%), linalyl formate (26.96%), α-terpineol (10.63%), 2-hexanoylfuran (5.81%), geranyl acetate (4.13%), and neryl acetate (2.30%); the structural formulas of these compounds are presented in Figure 1.

### 2.2. Activity Assay of Phytotoxicity Mitigation of PEO

#### 2.2.1. The Mitigation Effect of PEO on the Phytotoxicity of FE

**Figure 2:**
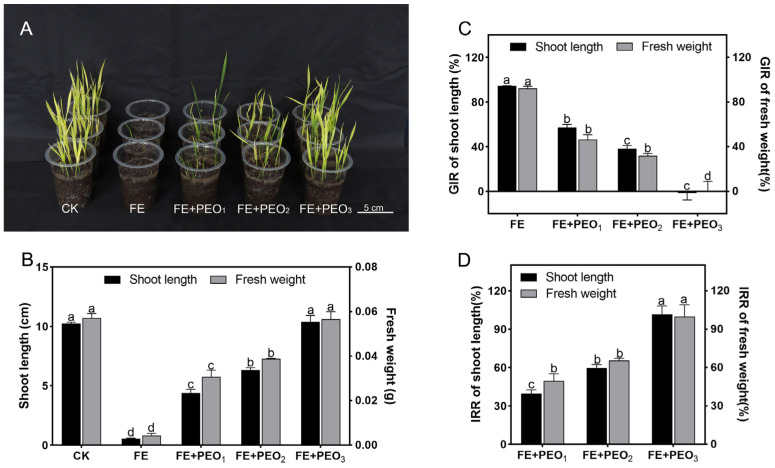
(**A**) Growth of rice seedlings under different treatments at 7 d. (**B**) Effects on rice shoot length and fresh weight of different treatments. (**C**) The growth inhibition rate (GIR) of shoot length and fresh weight of different treatments. (**D**) The injury recovery rate (IRR) of shoot length and fresh weight of different treatments, where FE is 0.8 mg/L, PEO_1_, PEO_2_, and PEO_3_ are 200, 400, and 800 mg/L perilla leaf essential oil (PEO), respectively. For each treatment, the means (±SE; *n* = 3) that are accompanied by distinct letters indicate a statistically significant difference at *p* < 0.05.

The results showed that PEO could alleviate the phytotoxicity of FE in rice seedlings (Figure 2A,B). At 0.8 mg/L of FE alone treatment, the growth of rice seedlings was seriously inhibited, and the growth inhibition rate (GIR) of shoot length and fresh weight were 94.43% and 92.34%, respectively (Figure 2C). Following the co-application of PEO at concentrations of 200, 400, and 800 mg/L, the mitigation effect on phytotoxicity increased with the concentration of PEO, peaking at 800 mg/L, and there were no significant differences in shoot length and fresh weight compared to the CK treatment. The IRR of shoot length and fresh weight with the co-application of 200 mg/L PEO was 39.58% and 49.96%, respectively, the IRR of co-application of 400 mg/L PEO was 59.47% and 65.38%, respectively, and the IRR of co-application of 800 mg/L PEO was 101.51% and 99.05%, respectively (Figure 2D).

#### 2.2.2. The Effect of PEO on the Phytotoxicity of Seven Herbicides

**Figure 3:**
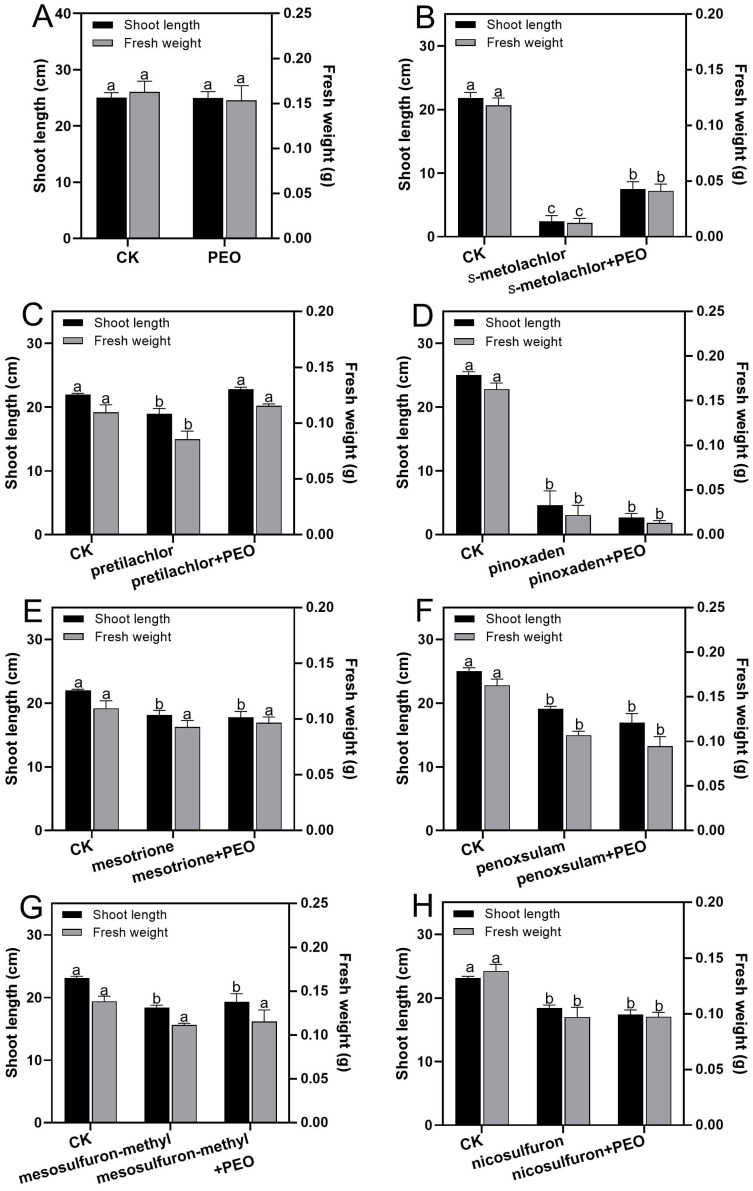
The mitigation activity of PEO against the phytotoxicity of seven herbicides. (**A**) PEO (800 mg/L) alone. (**B**) *s*-Metolachlor (30 mg/L). (**C**) Pretilachlor (1000 mg/L). (**D**) Pinoxaden (0.4 mg/L). (**E**) Mesotrione (400 mg/L). (**F**) Penoxsulam (200 mg/L). (**G**) Mesosulfuron-methyl (4 mg/L). (**H**) Nicosulfuron (5 mg/L). There were three treatment groups in B-I: CK, herbicide alone, and herbicide combined with 800 mg/L PEO treatment. For each treatment, the means (±SE; *n* = 3) that are accompanied by distinct letters indicate a statistically significant difference at *p* < 0.05.

The findings demonstrated that the co-application of 800 mg/L PEO may mitigate the damage caused by various types of herbicides to differing extents (Figure 3). The data of the effect of PEO on the phytotoxicity of seven herbicides are presented in Table S1(1-8) in the Supplementary Materials. The application of 800 mg/L of PEO alone did not produce a statistically significant impact on the shoot length and fresh weight of rice seedlings in comparison to the CK (Figure 3A). The phytotoxicity of *s*-metolachlor was alleviated by PEO (Figure 3B). The GIR of shoot length and fresh weight of 30 mg/L of *s*-metolachlor alone treatment was 88.77% and 89.48%, respectively, and the IRR of shoot length and fresh weight of *s*-metolachlor + PEO treatment was 26.07% and 27.34%, respectively. The phytotoxicity of pretilachlor was alleviated by PEO (Figure 3C). The GIR of shoot length and fresh weight of 1000 mg/L of pretilachlor alone treatment was 13.76% and 21.96%, respectively. The IRR of shoot length and fresh weight of pretilachlor + PEO treatment was 127.27% and 124.39%, respectively. Conversely, PEO had no significant effect on the phytotoxicity of pinoxaden (Figure 3D), mesotrione (Figure 3E), penoxsulam (Figure 3F), mesosulfuron-methyl (Figure 3G), and nicosulfuron (Figure 3H).

### 2.3. The Mitigation Effect of Main Components on the Phytotoxicity of FE

In order to identify the components with the highest ability to reduce phytotoxicity in PEO, the five components with a proportion greater than 2% were selected and tested for activity. These components include linalool, α-terpineol, 2-hexanoylfuran, geranyl acetate, and neryl acetate. Table 2 presents the IRR of rice shoot length and fresh weight following treatment with a mixture of concentrations of these components (50 mg/L) and FE (0.8 mg/L). The results showed that four components were able to reduce the damage to rice caused by FE. The IRR for shoot length and fresh weight was determined to be 73.17% and 73.02%, respectively, following treatment with 50 mg/L of 2-hexanoylfuran. The IRR for shoot length and fresh weight was determined to be 72.32% and 60.56%, respectively, under the treatment of 50 mg/L geranyl acetate mixed application, and 65.28% and 58.11%, respectively, under the treatment of 50 mg/L neryl acetate mixed application. In conclusion, 2-hexanoylfuran was the most active component to alleviate herbicide damage in PEO, with geranyl acetate and neryl acetate following.

### 2.4. The Mitigation Ability of 2-Hexanoylfuran to Phytotoxicity of FE

To assess the potential of 2-hexanofuran to mitigate the damage caused by FE in rice, this trial employed GR_50_ values to assess the influence of 2-hexanofuran on the dose–response relationship of rice subjected to the FE treatment. The results of the whole plant bioassay showed that 2-hexanoylfuran (50 mg/L) could significantly mitigate phytotoxicity in rice seedlings (Table 3). The GR_50_ value for rice shoot length was determined to be 0.38 mg/L in the FE treatment group, while it was found to be 2.04 mg/L in the FE + 2-hexanoylfuran treatment group. The inclusion of 2-hexanoylfuran resulted in a 5.32-fold increase in the tolerance of rice shoot length to FE. Moreover, the GR_50_ value for rice fresh weight was determined to be 0.40 mg/L in the FE treatment alone group and 2.13 mg/L in the FE + 2-hexanoylfuran treatment group, and the inclusion of 2-hexanoylfuran resulted in a 5.35-fold increase in the tolerance of rice fresh weight to FE.

## 3. Discussion

FE is a highly effective herbicide in rice fields, but it can easily cause etiolation, growth inhibition, and even death of rice seedlings [23,24]. In our study, a similar phytotoxicity symptom of FE was observed in rice seedlings after treatment with FE. The growth and development of rice seedlings were significantly inhibited after the application of 0.8 mg/L of FE. After 7 d of treatment, the FE group exhibited over 90% growth inhibition in rice seedlings, significantly impeding their normal development.

The application of safeners is the primary method for mitigating herbicide damage [25]. Among these, natural products have been extensively researched as safeners because of their beneficial biological activity and environmentally friendly characteristics [5,6,26]. In recent years, researchers have identified a variety of natural compounds in plant extracts that have the ability to mitigate phytotoxicity. For example, sanshool, the compound found in Szechuan pepper extracts, is effective in alleviating the damage caused by *s*-metolachlor in rice plants [27]. In this study, we found that PEO could protect rice from the damaging of FE for the first time.

The detoxification mechanisms of safeners exhibit both selectivity and specificity [28]. The natural safener can effectively mitigate the damage caused by various herbicides on the same crop, as well as reduce the phytotoxicity of a specific herbicide across different crops [29]. In this study, the ability of PEO to mitigate the phytotoxicity effects of various herbicides was assessed, and the results indicated that the mitigation effect of PEO on herbicide damage was specific. PEO demonstrated a targeted mitigation effect on the amide herbicides *s*-metolachlor and pretilachlor, as well as the APP herbicide FE, but it did not exhibit a mitigation effect on the new phenylpyrazoline herbicide pinoxaden, the triketone herbicide mesotrione, the triazolopyrimidine sulfonamide herbicide penoxsulam, or the sulfonylurea herbicides mesosulfuron-methyl and nicosulfuron. This suggests that the variation in phytotoxicity mitigation of PEO against different herbicides may be attributed to the differences in the chemical structures and mechanisms of action of the herbicides.

In addition, plant essential oils contain a diverse array of secondary metabolites with varying molecular structures, primarily including terpenes, terpenoids, phenylpropanoids, aldehydes, esters, alcohols, and ketones [30]. Similarly, PEO consists of multiple active compounds [10]. In this study, a total of 23 compounds were screened by GC–MS in PEO, of which linalool, α-terpineol, linalyl formate, 2-hexanoylfuran, neryl acetate, and geranyl acetate each accounted for more than 2%. In previous studies, perilla ketone, β-caryophyllene, linalool, and 2-hexanoylfuran were identified in PEO as the main components [31,32,33,34]. Therefore, the differences in secondary metabolites found in plant essential oils may vary based on the growth environment, extraction location, and extraction method of the plants [32,33,35,36].

The biological activity of plant essential oils is intricately associated with a diverse array of secondary metabolites [8,30]. In addition, these secondary metabolites can exhibit diverse biological activities, and there may also be synergistic or antagonistic interactions among different secondary metabolites [37]. In order to identify the secondary metabolites with the highest phytotoxicity mitigation activity in PEO, the phytotoxicity mitigation capabilities of six primary components were evaluated. The results indicated that 2-hexanoylfuran was the most effective, followed by geranyl acetate and neryl acetate. In addition, the neral derivatives of neryl acetate, as a plant-derived safener, have been shown to alleviate injury of FE in rice [23]. These studies demonstrate the potential of neryl acetate to be utilized as a natural safener.

In previous studies, 2-hexanoylfuran was primarily identified as an extract of *P. frutescens* [33]. This compound exhibits notable insecticidal activity against *Culex pipiens pallens*, demonstrating larvicidal and ovicidal effects, as well as repellency and oviposition deterrence [38]. However, the biological activities of other aspects of 2-hexanoylfuran are less well documented. Safeners can enhance the application of herbicides by increasing crop tolerance to these herbicides. The application of isoxadifen-ethyl hydrolysate, which is the safener for FE, increased the tolerance of rice to FE by 3.23 times [24]. In this study, the administration of 50 mg/L of 2-hexanoylfuran enhanced the tolerance of rice to FE by over 5 times. The compound 2-hexanofuran demonstrated a similar ability to mitigate phytotoxicity compared to isoxadifen-ethyl hydrolysate. In the development and commercialization of natural safeners, structurally modifying natural products that possess phytotoxicity mitigation properties as lead compounds can enhance their stability and biological activity, while also reducing production costs [39]. Therefore, 2-hexanoylfuran can serve as a novel lead compound for natural safeners, enhancing its potential applications through structural modifications.

## 4. Materials and Methods

### 4.1. Materials and Chemicals

Rice seeds of the 9311 variety were provided by the College of Agriculture, Guangxi University (Nanning, China). PEO was supplied by Ji’an Zhongxiang Natural Plant Co., Ltd., Ji’an, China. FE (TC, 95.8%) was purchased from Suzhou Marif Biology Co., Ltd., Suzhou, China. Linalool (98%) and neryl acetate (98%) were purchased from Beijing InnoChem Science & Technology Co., Ltd., Beijing, China. α-Terpineol (98%) and geranyl acetate (95%) were purchased from Shanghai Aladdin Biochemical Technology Co., Ltd., China. The compound 2-hexanoylfuran (99%) was purchased from Thermo Fisher Scientific Inc., Waltham, MA, USA. Tween 80 was purchased from Shanghai Macklin Biochemical Co., Ltd., Shanghai, China. FE(TC), PEO, linalool, α-terpineol, geranyl acetate, neryl acetate, and 2-hexanoylfuran were dissolved in DMF, Tween 80 (2% *v*/*v*), and then diluted to various concentrations with distilled water.

The 96% *s*-metolachlor (EC) was purchased from Syngenta (Suzhou) Crop Protection Co., Ltd., Suzhou, China. The 30% pretilachlor (EC) was purchased from Xianglin Mefront Biotechnology (Huai‘an) Co., Ltd., Huaian, China. The 2.5% penoxsulam (OD) was purchased from Jiangsu Futian Agrochemical Co. Ltd., Nanjing, China. The 10% mesotrione (OD) was purchased from Jiangsu Fengshan Biochemical Technology Co. Ltd., Yancheng, China. The 5% pinoxaden (EC) was purchased from Syngenta (Suzhou) Crop Protection Co., Ltd., Suzhou, China. The 4% nicosulfuron (OD) was purchased from Hebei Zhongbao Lvnong Crop Technology Co. LTD., Langfang, China. The 3% mesosulfuron-methyl (OD) was purchased from Henan Hansi Crop Protection Co. Ltd., Shangqiu, China. These seven herbicides were diluted to various concentrations with distilled water. All other reagents were of analytical-grade purity.

### 4.2. Rice Cultivation and Growth Bioassays

Rice seeds that had been soaked in distilled water for 24 h were then placed in a dark environment and incubated at 28 °C for 48 h to stimulate germination. The rice seeds with a coleoptile length of 0.5 cm were selected and immersed in 40 mL of the treatment solution for 1 h. The treated seeds were washed four times with distilled water and then planted in plastic cups (7 cm in diameter, 8 cm in height) filled with Pindstrup substrate. The cups were placed in a growth chamber with a 12 h light and 12 h dark photocycle, with day and night temperatures set at 28 °C and 25 °C, and a light intensity of 12,000 lx. Each treatment included eight seeds and was replicated three times. Shoot length and fresh weight of rice seedlings were measured at 7 d after treatment. Subsequently, the growth inhibition rate (GIR), injury recovery rate (IRR), doses causing a 50% reduction in growth (GR_50_), and resistance index (RI) were calculated according to the previously reported methods [24,40].

### 4.3. Assay Methods for Mitigation Activity of Phytotoxicity

#### 4.3.1. Determination of the Mitigation Activity of PEO Against the Phytotoxicity of FE

The treatment doses were as follows: FE (0.8 mg/L), FE + PEO_1_ (0.8 mg/L + 200 mg/L), FE + PEO_2_ (0.8 mg/L + 400 mg/L), and FE + PEO_3_ (0.8 mg/L + 800 mg/L). Distilled water with a 2% Tween 80 solvent formulation was used as the CK treatment.

#### 4.3.2. Determination of the Mitigation Activity of PEO Against the Phytotoxicity of Eight Herbicides

All herbicide concentrations utilized in this trial represented the concentrations of the active ingredients. The treatment doses were as follows: PEO (800 mg/L), *s*-metolachlor (30 mg/L), *s*-metolachlor + PEO (30 mg/L + 800 mg/L), pretilachlor (1000 mg/L), pretilachlor + PEO (1000 mg/L + 800 mg/L), penoxsulam (200 mg/L), penoxsulam + PEO (200 mg/L + 800 mg/L), pinoxaden (0.4 mg/L), pinoxaden + PEO (0.4 mg/L + 800 mg/L), mesotrione (400 mg/L), mesotrione + PEO (400 mg/L + 800 mg/L), mesosulfuron-methyl (4 mg/L), mesosulfuron-methyl + PEO (4 mg/L + 800 mg/L), nicosulfuron (5 mg/L), and nicosulfuron + PEO (5 mg/L + 800 mg/L). Distilled water was used as the CK treatment.

#### 4.3.3. Determination of the Mitigation Activity of Six Compounds Against the Phytotoxicity of FE

The treatment doses were as follows: FE (0.8 mg/L), FE + linalool (0.8 mg/L + 50 mg/L), FE + α-terpineol (0.8 mg/L + 50 mg/L), FE + geranyl acetate (0.8 mg/L + 50 mg/L), FE + neryl acetate (0.8 mg/L + 50 mg/L), and FE + 2-hexanoylfuran (0.8 mg/L + 50 mg/L). Distilled water with a 2% Tween 80 solvent formulation was used as the CK treatment.

#### 4.3.4. Effect of 2-Hexanoylfuran on FE Tolerance in Rice

The FE doses (0.125, 0.25, 0.5, 1, 2 mg/L) were used alone, and the FE doses (0.125, 0.25, 0.5, 1, 2, 4, 8, 16 mg/L) were used in combination with the 50 mg/L 2-hexanoylfuran. Distilled water with a 2% Tween 80 solvent formulation was used as the CK treatment.

### 4.4. Chemical Analysis of PEO

The methods for GC–MS were conducted in accordance with the reported methods, with appropriate modifications made as necessary [41]. GC–MS analysis was performed on a GCMS-TQ8030 Gas Chromatograph Mass Spectrometer (Shimadzu, Kyoto, Japan). A fused silica capillary Restek Rtx-5 (5% phenyl methyl siloxane) column (30.0 m × 0.25 mm × 0.25 μm) was used for separation. EI (electron impact) was used as the ion source, which had a temperature of 280 °C. The sector mass analyser was set to scan from 33 to 450 amu (*m*/*z*). The identification of the components was conducted by comparing their recorded mass spectra with the standard mass spectra available in the National Institute of Standards and Technology (NIST 11) library. The relative percentage composition of each component in PEO was determined utilizing the peak area normalization method in GC–MS. The essential oil samples were diluted 50 times with acetone and subsequently analysed using the machine.

### 4.5. Statistical Analysis

All data are expressed as the mean ± standard error (SE) derived from three replicates. Statistical analyses were conducted using SPSS 20.0 (SPSS Inc., Chicago, IL, USA), employing one-way ANOVA followed by Duncan’s multiple range test with a significance level set at *p* < 0.05. The figures were created utilizing GraphPad Prism 9.

## 5. Conclusions

This study is the first to demonstrate the ability of PEO to alleviate the damage caused by FE in rice. PEO showed specific mitigation of herbicide damage, and it also had mitigation activity against *s*-metolachlor and pretilachlor. Linalool, linalyl formate, α-terpineol, 2-hexanoylfuran, geranyl acetate, and neryl acetate were the primary components. Among them, 2-hexanoylfuran, geranyl acetate and neryl acetate were the active compounds with the strongest mitigating activity in PEO. Our study offers a valuable reference for a more comprehensive understanding of the biological activities of PEO and 2-hexanoylfuran. Additionally, it can broaden the application range of *P. frutescens* and its extracts, thereby enhancing the market value of *P. frutescens.*

## Figures and Tables

**Table 1:** Chemical composition of PEO.

No.	Retention Time	Relative Content (%)	Constituents	CAS No.
1	6.100	1.09%	β-Pinene	127-91-3
2	6.838	0.54%	Limonene	5989-27-5
3	6.883	0.66%	*trans*-β-ocimene	3779-61-1
4	7.065	0.97%	*cis*-β-ocimene	3338-55-4
5	7.769	0.45%	Terpinolene	586-62-9
6	7.960	36.49%	Linalool	78-70-6
7	9.373	10.63%	α-Terpineol	10482-56-1
8	9.650	1.22%	Nerol	106-25-2
9	9.873	26.96%	Linalyl formate	115-99-1
10	9.912	1.48%	Geraniol	106-24-1
11	9.943	5.81%	2-Hexanoylfuran	14360-50-0
12	10.459	1.18%	Unknown	-
13	10.917	2.30%	Neryl acetate	141-12-8
14	11.082	4.13%	Geranyl acetate	105-87-3
15	11.171	0.35%	α-Cubebene	17699-14-8
16	11.256	0.36%	β-Bourbonene	5208-59-3
17	11.428	0.19%	Caryophyllene	13877-93-5
18	11.564	1.49%	β-Caryophyllene	87-44-5
19	11.847	0.14%	α-Caryophyllene	6753-98-6
20	11.951	0.29%	α-Bergamotene	17699-05-7
21	12.024	0.43%	β-copaene	317819-78-6
22	12.241	0.25%	δ-Cadinene	483-76-1
23	12.771	0.49%	Caryophyllene oxide	1139-30-6
24	13.983	1.22%	Isopropyl myristate	110-27-0
25	14.635	0.87%	Unknown	-
	Total	100%		

**Table 2:** The mitigation activities of five components on rice FE phytotoxicity.

Compound	IRR (Shoot Length) (%)	IRR (Fresh Weight) (%)
2-Hexanoylfuran	73.17 ± 5.31 a	73.02 ± 4.86 a
Geranyl acetate	72.32 ± 6.38 a	60.56 ± 2.92 b
Neryl acetate	65.28 ± 2.28 a	58.11 ± 1.03 b
Linalool	31.36 ± 1.89 b	37.81 ± 1.64 c
α-Terpineol	13.49 ± 0.94 c	19.69 ± 0.90 d

Notes: Compounds (50 mg/L) were mixed with FE (0.8 mg/L), and shoot length and fresh weight were assessed following a treatment duration of 7 d, and the IRR was calculated compared with the FE (0.8 mg/L) alone treatment group. For each treatment, the means (±SE; *n* = 3) that are accompanied by distinct letters indicate a statistically significant difference at *p* < 0.05.

**Table 3:** The effect of 2-hexanoylfuran on the tolerance of rice plants to FE.

Treatments ^a^	Target	GR_50_ (mg/L) ^b^	Regression Equation	R^2 c^	RI ^d^
FE	Shoot length	0.38	y = 2.6262x + 6.0946	0.9795	
	Fresh weight	0.40	y = 2.5943x + 6.035	0.9767	
FE + 2-hexanoylfuran	Shoot length	2.04	y = 1.4445x + 4.5536	0.9799	5.32
	Fresh weight	2.13	y = 1.4241x + 4.5313	0.9808	5.35

^a^ FE, 0.125–2 mg/L of FE alone treatments; FE + 2-hexanoylfuran, 0.125–16 mg/L of FE co-application with 50 mg/L of 2-hexanoylfuran treatments. ^b^ GR_50_, the herbicide concentration causing a 50% growth injury of plants. ^c^ The coefficient of determination (R^2^) and the regression equation were obtained from regression analysis. ^d^ Resistance levels are indicated by the resistance index (RI). RI = GR_50_ (FE + 2-hexanoylfuran)/GR_50_ (FE).

## Data Availability

The data are contained in this article.

## Supplementary Materials

The following supporting information can be downloaded at: https://www.mdpi.com/article/10.3390/plants13202946/s1, Table S1-1: The effect of perilla leaf essential oil (PEO) treatment alone on rice growth; Table S1-2: The mitigation activity of PEO against the phytotoxicity of s-metolachlor; Table S1-3: The mitigation activity of PEO against the phytotoxicity of pretilachlor; Table S1-4: The mitigation activity of PEO against the phytotoxicity of pinoxaden; Table S1-5: The mitigation activity of PEO against the phytotoxicity of mesotrione; Table S1-6: The mitigation activity of PEO against the phytotoxicity of penoxsulam; Table S1-7: The mitigation activity of PEO against the phytotoxicity of mesosulfuron-methyl; Table S1-8: The mitigation activity of PEO against the phytotoxicity of penoxsulam; Figure S1: The total ions chromatograph map of perilla leaf essential oil (PEO); Figure S2-1: The MS spectra of compound 1; Figure S2-2: The MS spectra of compound 2; Figure S2-3: The MS spectra of compound 3; Figure S2-4: The MS spectra of compound 4; Figure S2-5: The MS spectra of compound 5; Figure S2-6: The MS spectra of compound 6; Figure S2-7: The MS spectra of compound 7; Figure S2-8: The MS spectra of compound 8; Figure S2-9: The MS spectra of compound 9; Figure S2-10: The MS spectra of compound 10; Figure S2-11: The MS spectra of compound 11; Figure S2-12: The MS spectra of compound 13; Figure S2-13: The MS spectra of compound 14; Figure S2-14: The MS spectra of compound 15; Figure S2-15: The MS spectra of compound 16; Figure S2-16: The MS spectra of compound 17; Figure S2-17: The MS spectra of compound 18; Figure S2-18: The MS spectra of compound 19; Figure S2-19: The MS spectra of compound 20; Figure S2-20: The MS spectra of compound 21; Figure S2-21: The MS spectra of compound 22; Figure S2-22: The MS spectra of compound 23; Figure S2-23: The MS spectra of compound 24.
