# Supplementary material for: The Mitigating Effects of Perilla Leaf Essential Oil on the Phytotoxicity of Fenoxaprop-P-Ethyl in Rice Seedlings
Source: Plants (Basel). 2024 Oct 21;13(20):2946. doi: 10.3390/plants13202946 (PMC11510985; doi:10.3390/plants13202946)
Supplement: Supplementary file 1 [file plants-13-02946-s001.zip › plants-3237674-supplementary.pdf]

Supplementary Materials

The Mitigating Effects of Perilla Leaf Essential Oil on the Phytotoxicity of Fenoxaprop-P-ethyl in Rice Seedlings

Jiuying Li [1, 2, †], Yinghui Zhu [1, †], Lanlan Sun [1], Hongle Xu [1], Wangcang Su [1], Fei Xue [1], Chuantao Lu [1], Wenwei Tang [2], and Renhai Wu [1,*]

[1] Henan Key Laboratory of Crop Pest Control, Institute of Plant Protection, Henan Academy of Agricultural Sciences, Zhengzhou 450002, China.

[2] National Demonstration Center for Experimental Plant Science Education, Guangxi Key Laboratory of Agro-Environment and Agric-Product Safety, College of Agriculture, Guangxi University, Nanning 530004, China.

[3] Sumy National Agricultural University, Sumy 40000, Ukraine.

[*] Correspondence: renhai.wu@163.com

[†] These authors contributed equally to this work.

Table S1-1 The effect of perilla leaf essential oil (PEO) treatment alone on rice growth

Treatments	Shoot length (cm)	Fresh weight (g)
CK	25.04±0.52 a	0.1629±0.0069 a
PEO	24.97±0.66 a	0.1534±0.0094 a

Notes: The concentration of PEO was 800mg/L, and shoot length and fresh weight were assessed following a treatment duration of 7 d. For each treatment, the means (±SE; n = 3) that are accompanied by distinct letters indicate a statistically significant difference at $p < 0.05$.

Table S1-2 The mitigation activity of PEO against the phytotoxicity of *s*-metolachlor

Treatments	Shoot length (cm)	Fresh weight (g)
CK	21.85±0.82 a	0.1180±0.0066 a
s-metolachlor	2.45±0.85 c	0.0124±0.0040 c
s-metolachlor + PEO	7.51±1.14 b	0.0413±0.0060 b

Notes: The concentration of *s*-metolachlor was 30 mg/L, and the PEO was 800mg/L. The shoot length and fresh weight were assessed following a treatment duration of 7 d. For each treatment, the means (±SE; n = 3) that are accompanied by distinct letters indicate a statistically significant difference at $p < 0.05$.

Table S1-3 The mitigation activity of PEO against the phytotoxicity of pretilachlor

Treatments	Shoot length (cm)	Fresh weight (g)
CK	21.98±0.18 a	0.1097±0.0067 a
pretilachlor	18.95±0.83 b	0.0856±0.0072 b
pretilachlor + PEO	22.81±0.33 a	0.1155±0.0016 a

Notes: The concentration of pretilachlor was 1000 mg/L, and the PEO was 800mg/L. The shoot length and fresh weight were assessed following a treatment duration of 7 d. For each treatment, the means (±SE; n = 3) that are accompanied by distinct letters indicate a statistically significant difference at $p < 0.05$.

Table S1-4 The mitigation activity of PEO against the phytotoxicity of pinoxaden

Treatments	Shoot length (cm)	Fresh weight (g)
CK	25.04±0.52 a	0.1629±0.0069 a
pinoxaden	4.63±2.22 b	0.0218±0.0110 b
pinoxaden + PEO	2.70±0.60 b	0.0130±0.0026 b

Notes: The concentration of pinoxaden was 0.4 mg/L, and the PEO was 800mg/L. The shoot length and fresh weight were assessed following a treatment duration of 7 d. For each treatment, the means (±SE; n = 3) that are accompanied by distinct letters indicate a statistically significant difference at $p < 0.05$.

Table S1-5 The mitigation activity of PEO against the phytotoxicity of mesotrione

Treatments	Shoot length (cm)	Fresh weight (g)
CK	21.98±0.18 a	0.1097±0.0067 a
mesotrione	18.14±0.68 b	0.0928±0.0058 a
mesotrione + PEO	17.75±0.92 b	0.0967±0.0052 a

Notes: The concentration of mesotrione was 400 mg/L, and the PEO was 800mg/L. The shoot length and fresh weight were assessed following a treatment duration of 7 d. For each treatment, the means (±SE; n = 3) that are accompanied by distinct letters indicate a statistically significant difference at $p < 0.05$.

Table S1-6 The mitigation activity of PEO against the phytotoxicity of penoxsulam

Treatments	Shoot length (cm)	Fresh weight (g)
CK	25.04±0.52 a	0.1629±0.0069 a
penoxsulam	19.12±0.37 b	0.1071±0.0043 b
penoxsulam + PEO	16.95±1.42 b	0.0947±0.0107 b

Notes: The concentration of penoxsulam was 200 mg/L, and the PEO was 800mg/L. The shoot length and fresh weight were assessed following a treatment duration of 7 d. For each treatment, the means (±SE; n = 3) that are accompanied by distinct letters indicate a statistically significant

difference at $p < 0.05$.

Table S1-7 The mitigation activity of PEO against the phytotoxicity of mesosulfuron-methyl

Treatments	Shoot length (cm)	Fresh weight (g)
CK	23.12±0.28 a	0.1385±0.0059 a
mesosulfuron-methyl	18.40±0.37 b	0.1116±0.0019 a
mesosulfuron-methyl + PEO	19.36±1.24 b	0.1155±0.0131 a

Notes: The concentration of mesosulfuron-methyl was 4 mg/L, and the PEO was 800mg/L. The shoot length and fresh weight were assessed following a treatment duration of 7 d. For each treatment, the means (±SE; $n = 3$) that are accompanied by distinct letters indicate a statistically significant difference at $p < 0.05$.

Table S1-8 The mitigation activity of PEO against the phytotoxicity of penoxsulam

Treatments	Shoot length (cm)	Fresh weight (g)
CK	23.12±0.28 a	0.1385±0.0059 a
nicosulfuron	18.44±0.44 b	0.0970±0.0089 b
nicosulfuron + PEO	17.38±0.74 b	0.0975±0.00391 b

Notes: The concentration of nicosulfuron was 5 mg/L, and the PEO was 800mg/L. The shoot length and fresh weight were assessed following a treatment duration of 7 d. For each treatment, the means (±SE; $n = 3$) that are accompanied by distinct letters indicate a statistically significant difference at $p < 0.05$.

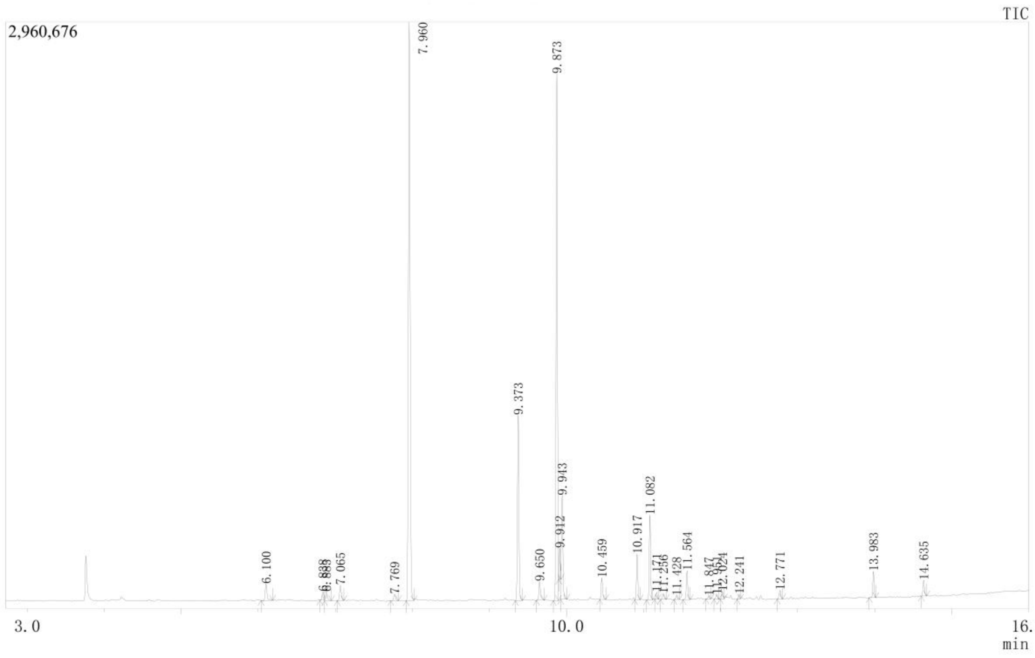

Figure S1. The total ions chromatograph map of perilla leaf essential oil (PEO).

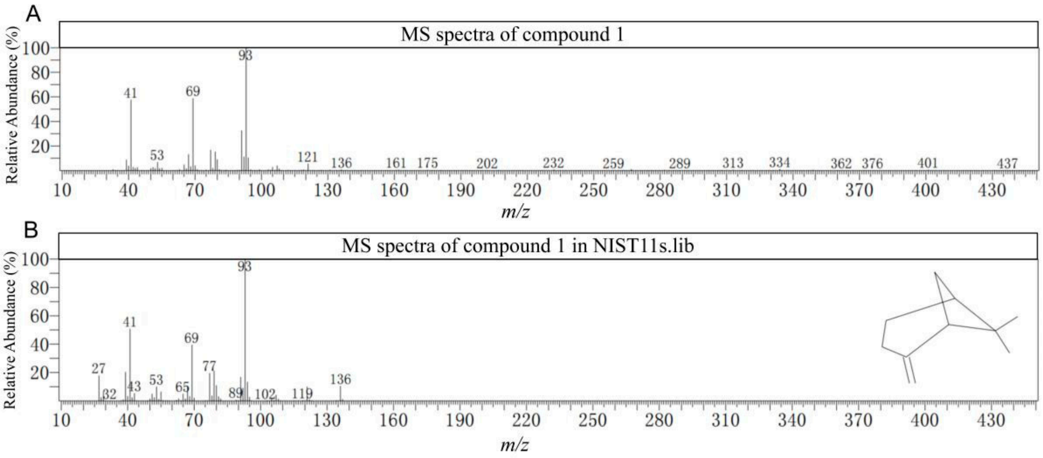

Figure S2-1. The MS spectra of compound 1.

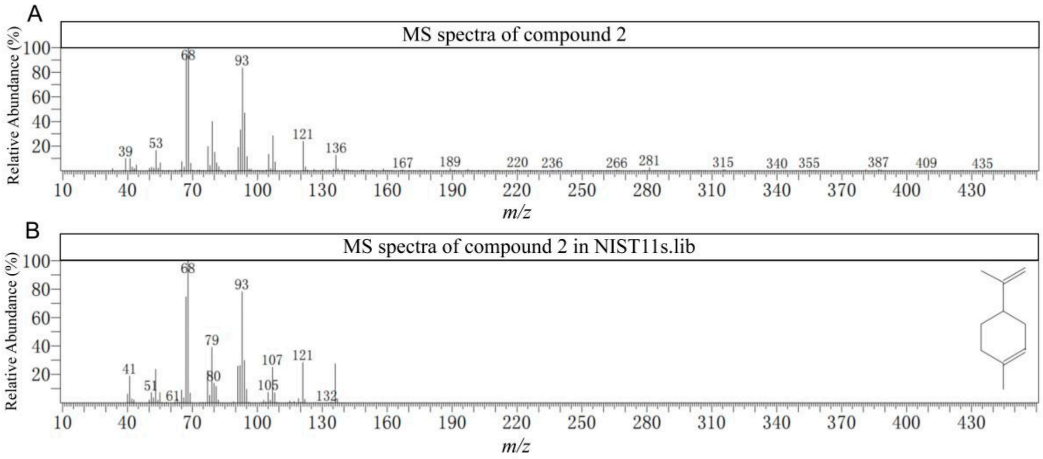

Figure S2-2. The MS spectra of compound 2.

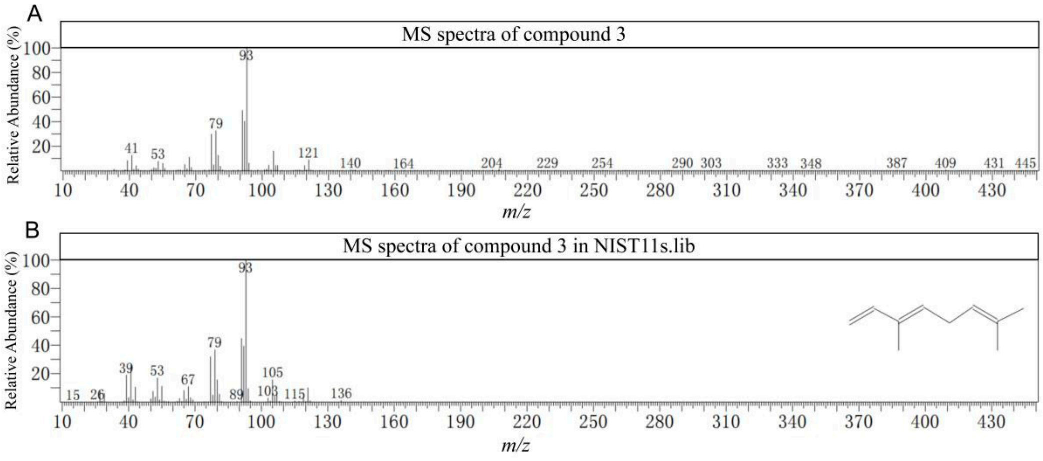

Figure S2-3. The MS spectra of compound 3.

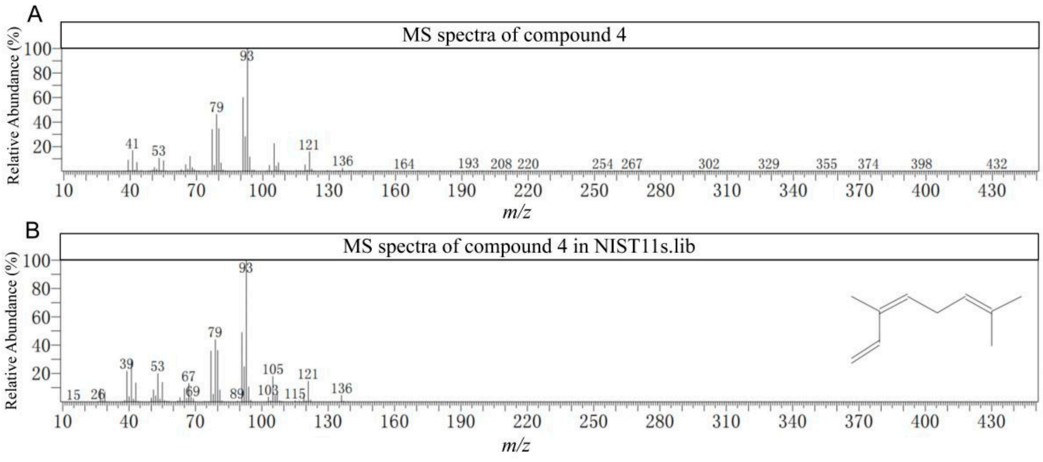

Figure S2-4. The MS spectra of compound 4.

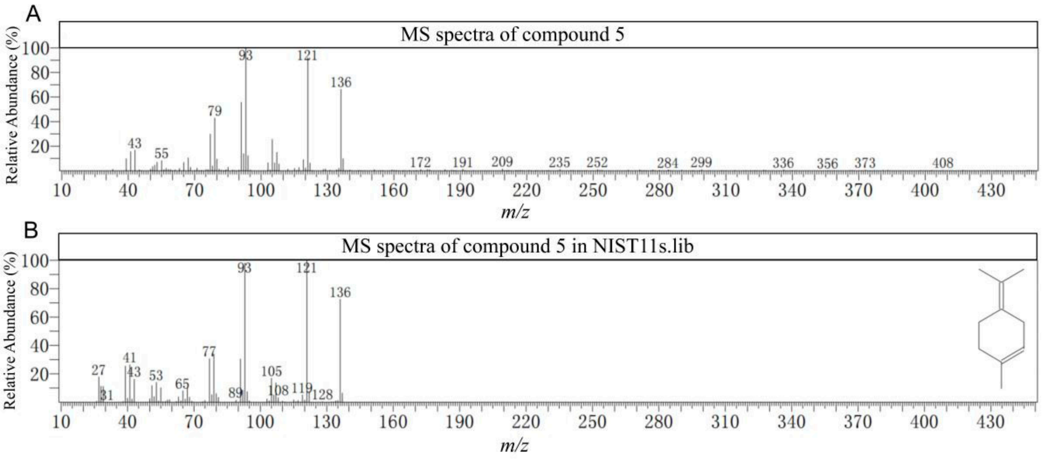

Figure S2-5. The MS spectra of compound 5.

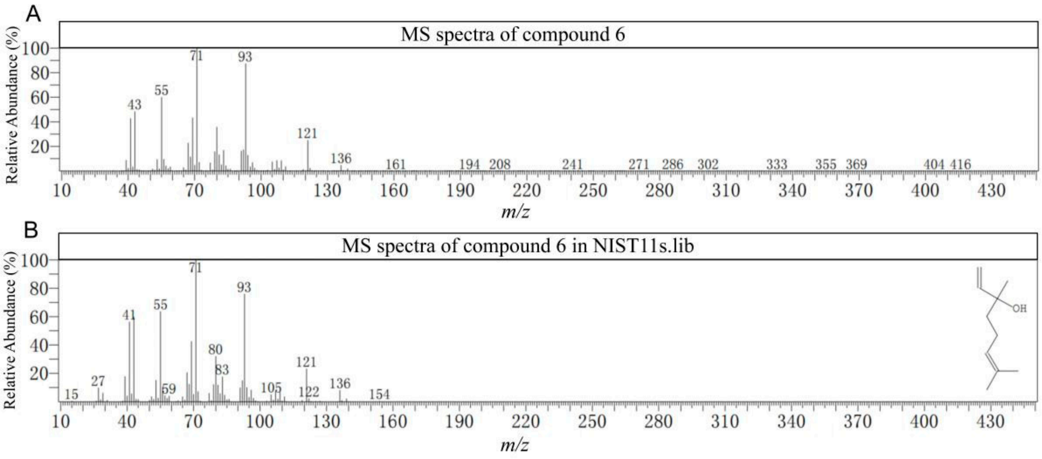

Figure S2-6. The MS spectra of compound 6.

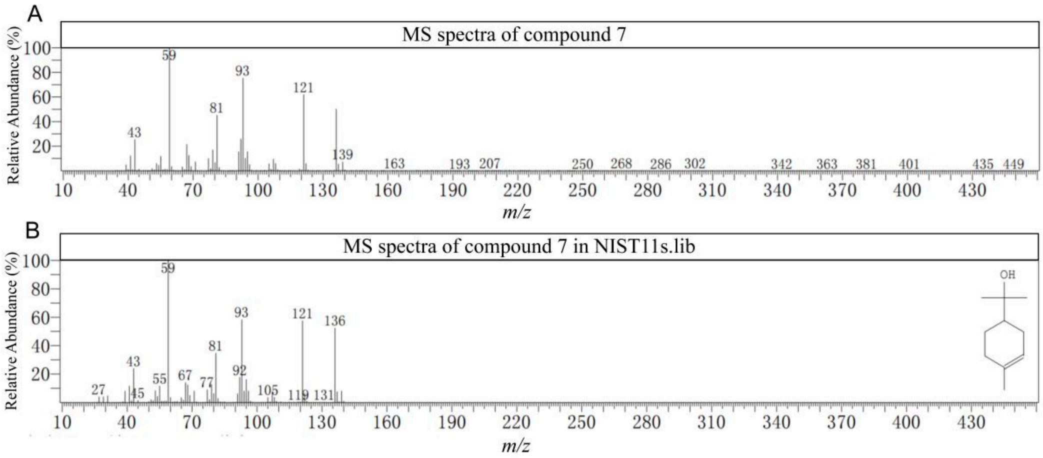

Figure S2-7. The MS spectra of compound 7.

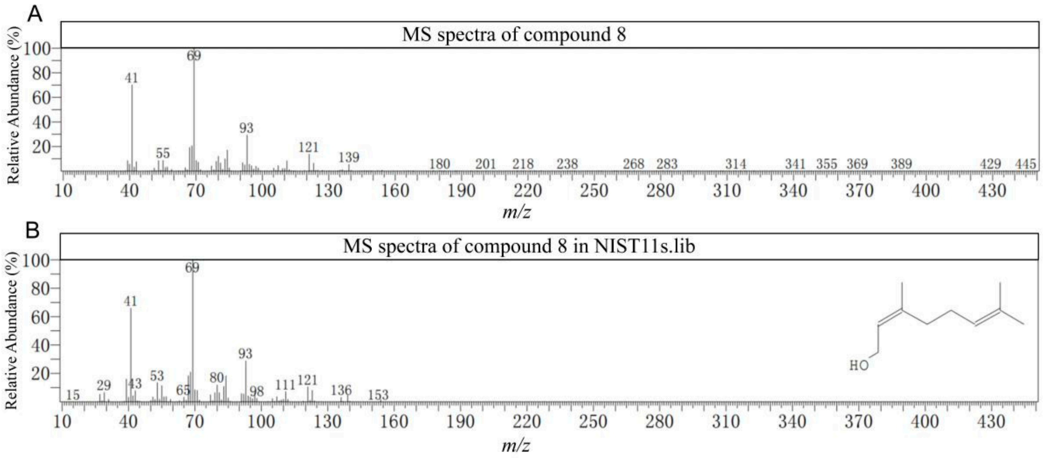

Figure S2-8. The MS spectra of compound 8.

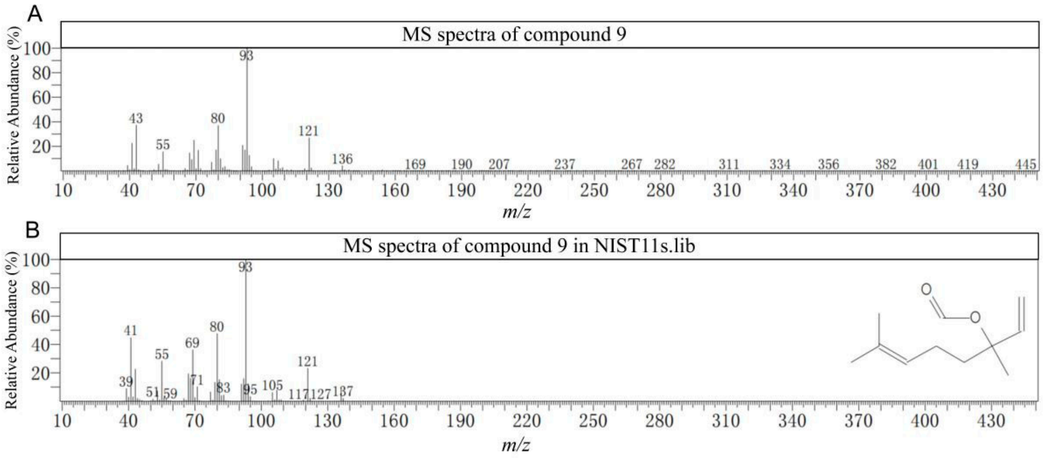

Figure S2-9. The MS spectra of compound 9.

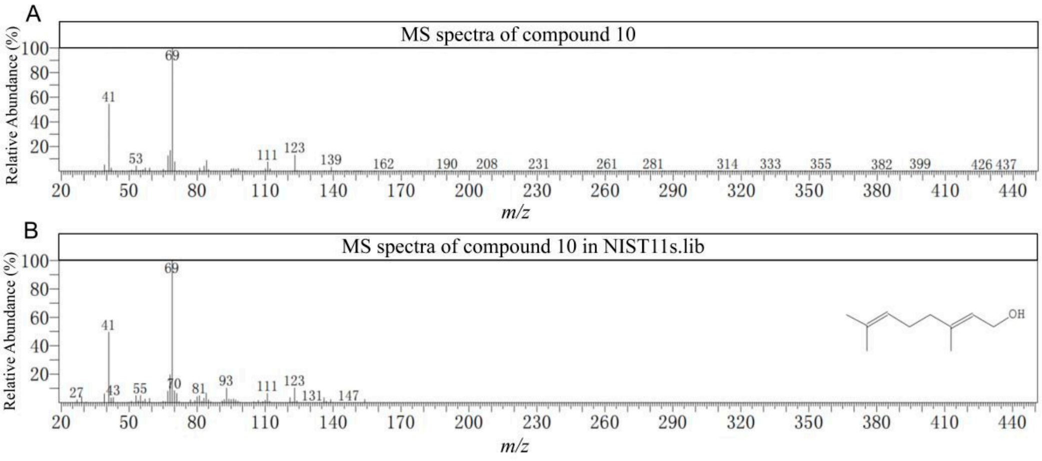

Figure S2-10. The MS spectra of compound 10.

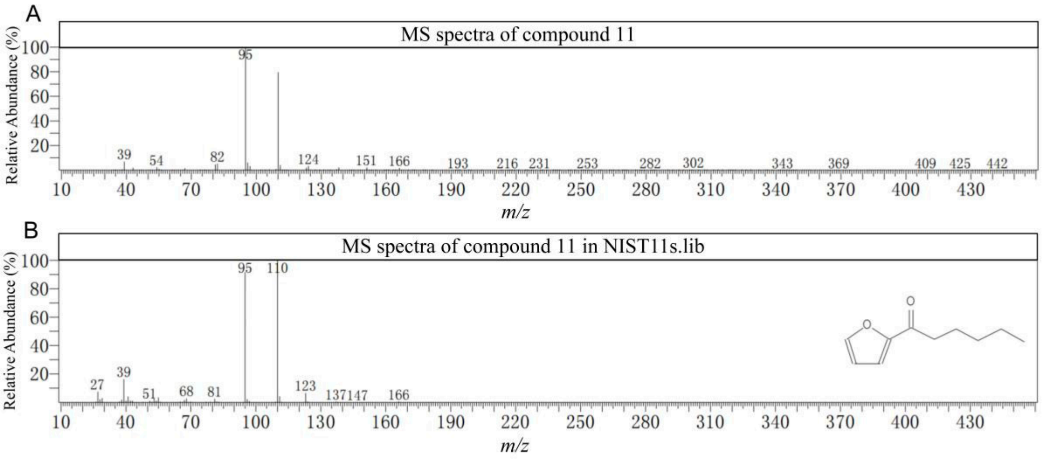

Figure S2-11. The MS spectra of compound 11.

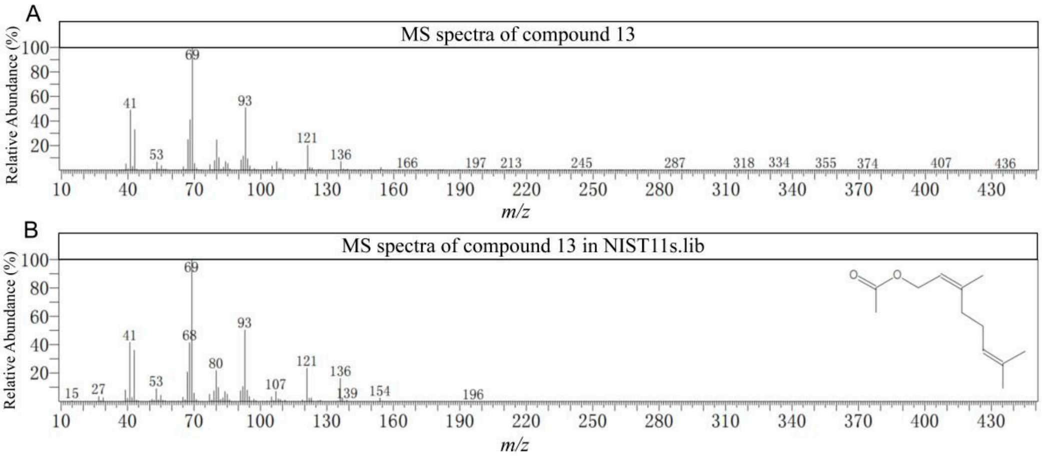

Figure S2-12. The MS spectra of compound 13.

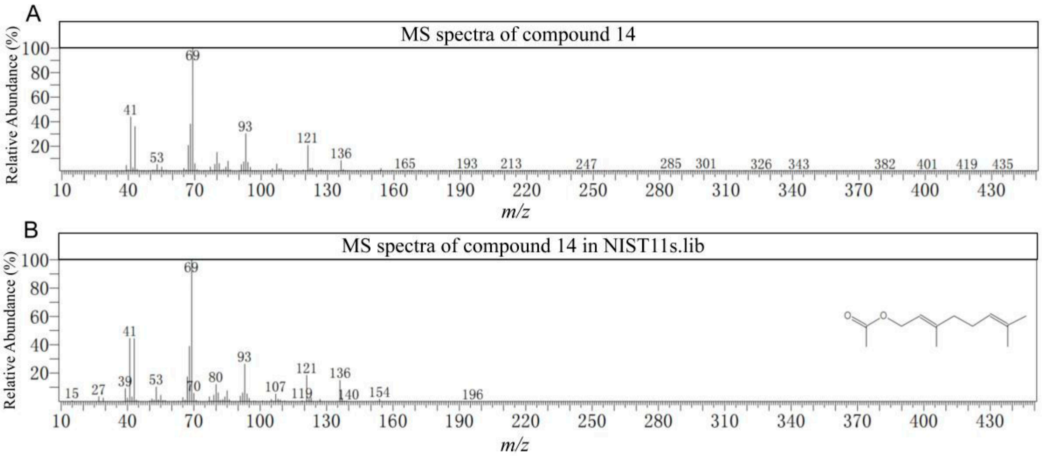

Figure S2-13. The MS spectra of compound 14.

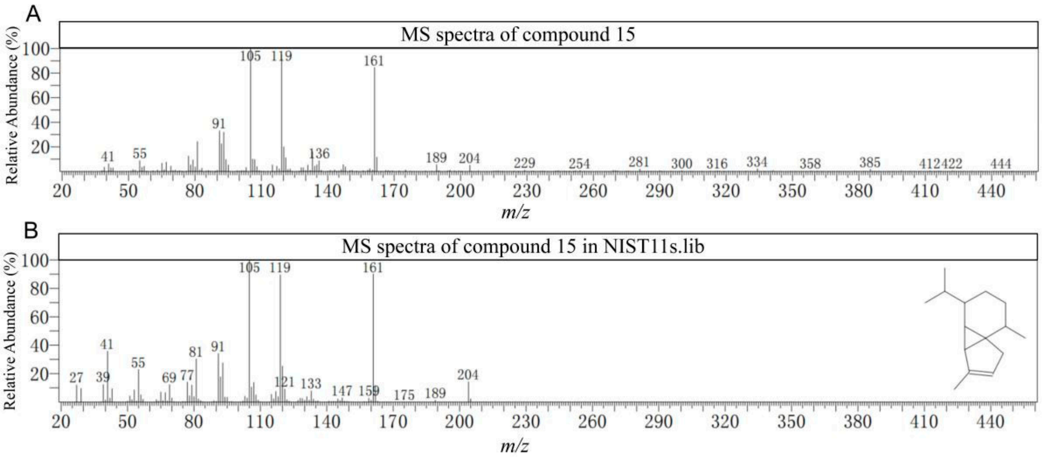

Figure S2-14. The MS spectra of compound 15.

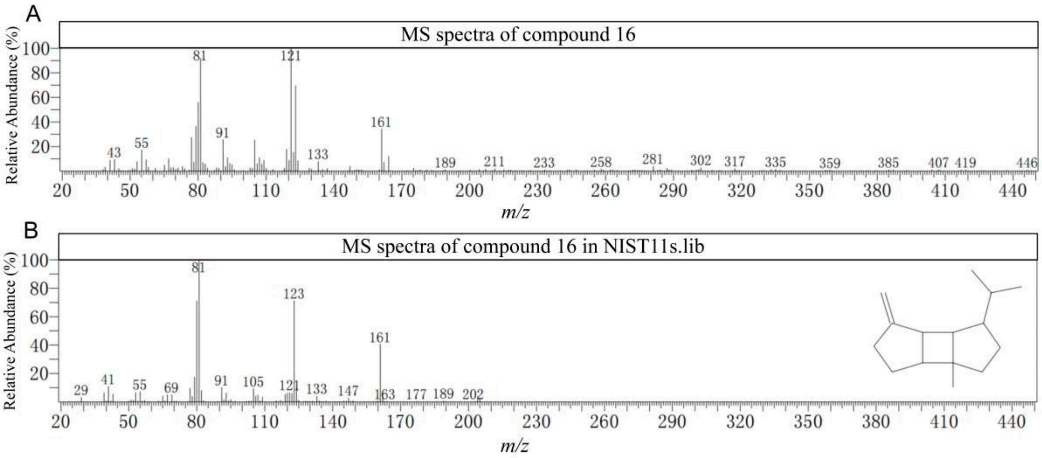

Figure S2-15. The MS spectra of compound 16.

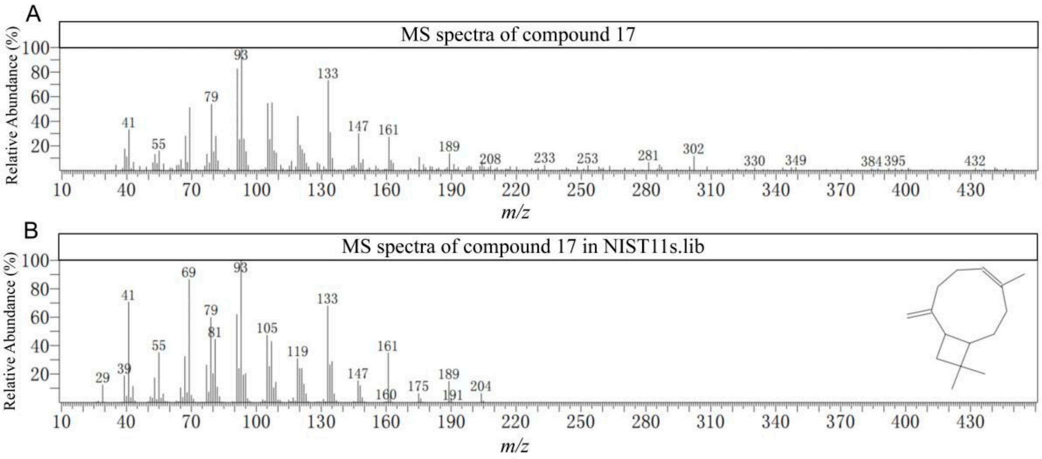

Figure S2-16. The MS spectra of compound 17.

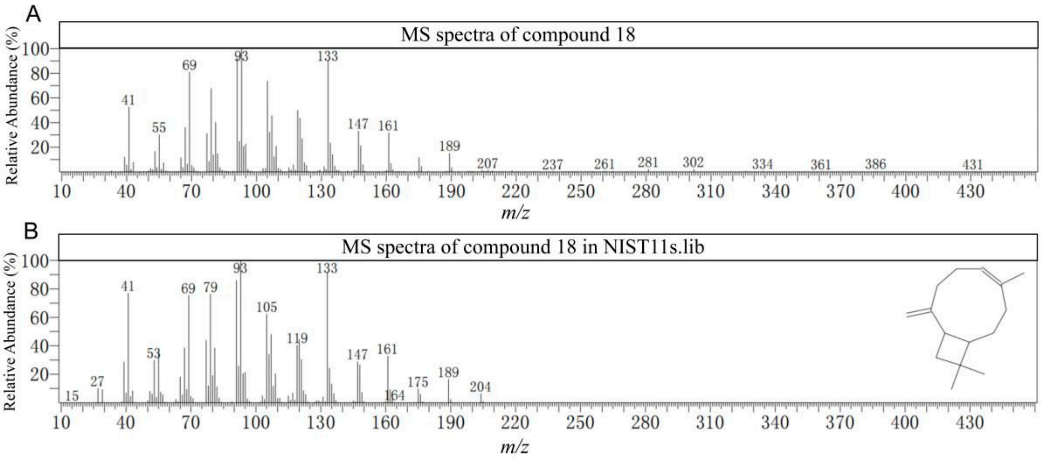

Figure S2-17. The MS spectra of compound 18.

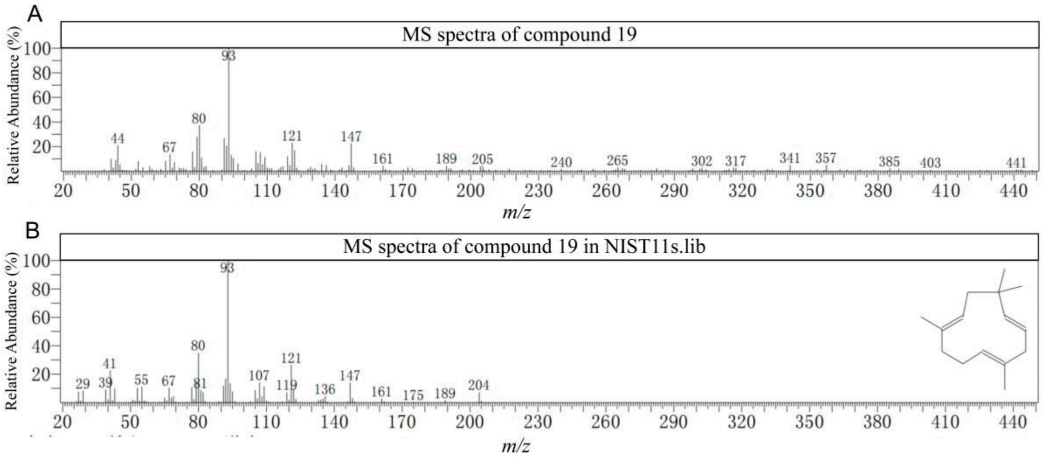

Figure S2-18. The MS spectra of compound 19.

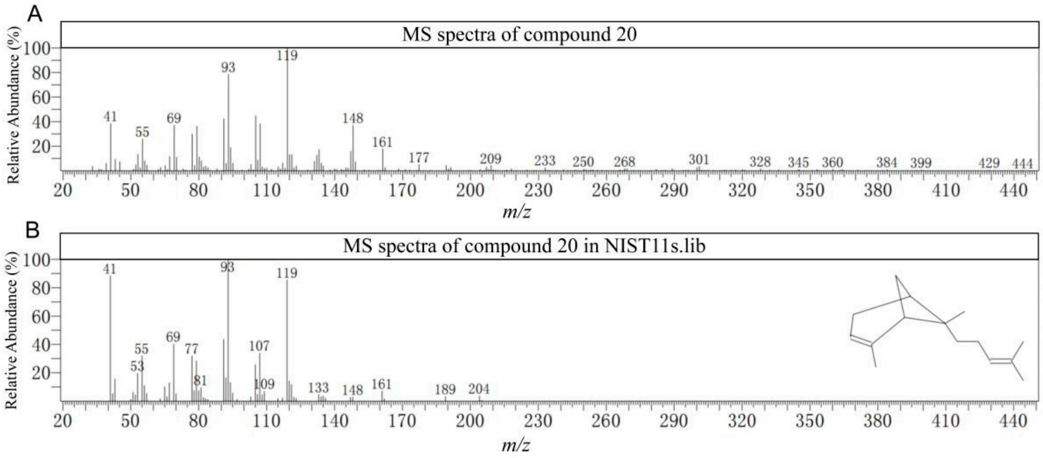

Figure S2-19. The MS spectra of compound 20.

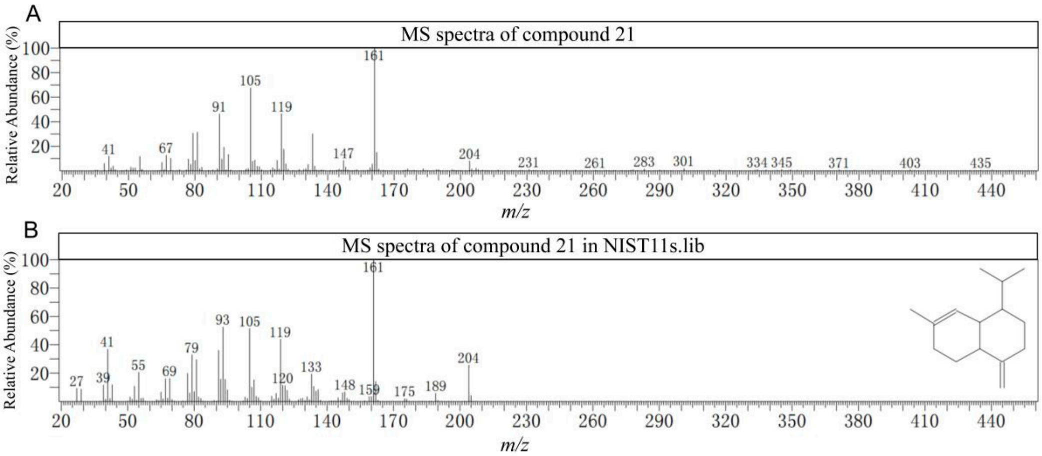

Figure S2-20. The MS spectra of compound 21.

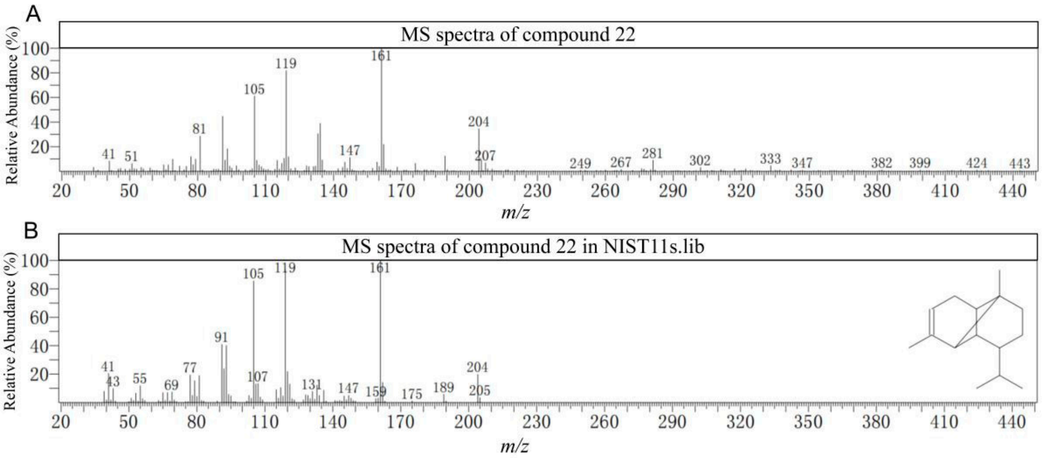

Figure S2-21. The MS spectra of compound 22.

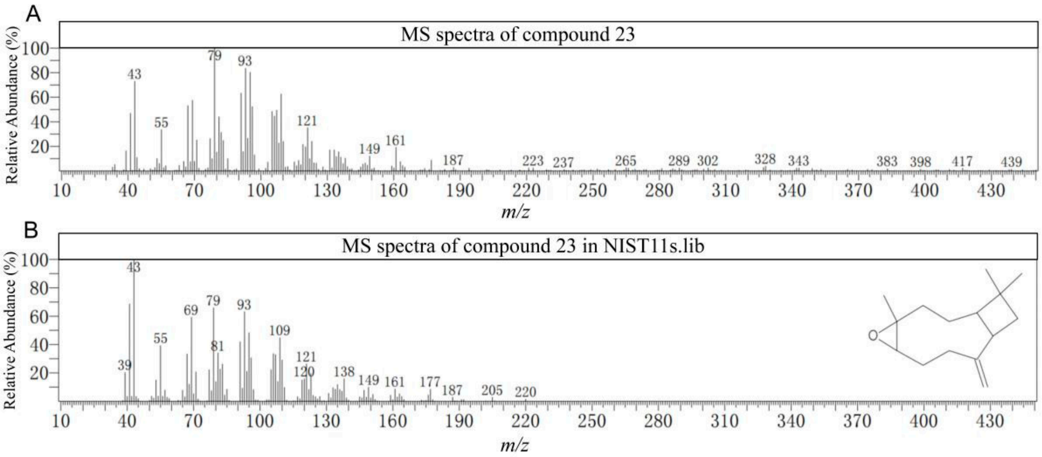

Figure S2-22. The MS spectra of compound 23.

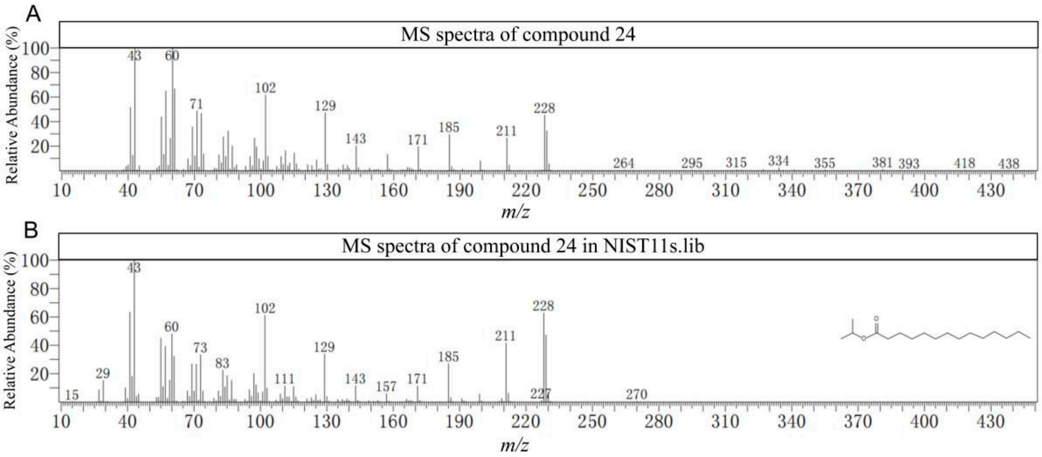

Figure S2-23. The MS spectra of compound 24.
